# Prevalence of chronic musculoskeletal disorders in elderly Brazilians: a systematic review of the literature

**DOI:** 10.1186/1471-2474-13-82

**Published:** 2012-05-29

**Authors:** Vivian S Miranda, Vivielle BF deCarvalho, Luciana AC Machado, João Marcos D Dias

**Affiliations:** 1 Department of Physiotherapy, Federal University of Minas Gerais, Avenida Presidente Antônio Carlos 6627 Pampulha, Belo Horizonte, MG 31.270-901, Brazil; 2 Faculty of Medicine, Federal University of Minas Gerais, Avenida Alfredo Balena 190, Santa Efigênia, Belo Horizonte, MG 30.190-100, Brazil

**Keywords:** Prevalence, Epidemiology, Chronic pain, Musculoskeletal disorders, Elderly

## Abstract

****Background**:**

Population ageing is a worldwide phenomenon that has recently challenged public healthcare systems. The knowledge of the burden of chronic musculoskeletal disorders in elders is still limited, particularly in the developing world. This systematic review aimed to investigate the prevalence of chronic musculoskeletal disorders in elderly Brazilians.

****Methods**:**

A comprehensive literature search was performed in five electronic databases (from inception to January 2012) and completed by additional searches in reference lists. Two review authors independently selected the eligible studies and extracted data on participants’ characteristics and rates of chronic musculoskeletal disorders. One review author extracted methodological quality data. We performed a critical synthesis of the results, which were grouped into the diagnoses “chronic musculoskeletal pain” or “specific musculoskeletal diagnoses”.

****Results**:**

Twenty five studies reporting on a total of 116,091 elderly Brazilians were included. Eight studies (32%) were of high methodological quality. There was a large variation in the measure of prevalence used by individual studies and in their definition of chronic pain. Prevalence estimates reached 86% for chronic musculoskeletal pain in any location. Studies investigating multiple pain sites found the lower limb and the spine to be the most prevalent complaints (50% each). Arthritis and rheumatism (including osteoarthritis) were the most prevalent specific musculoskeletal diagnoses (9% to 40%), followed by herniated disc (6% to 27%).

****Conclusions**:**

Despite the growth of the elderly population worldwide, high-quality research on the burden of chronic musculoskeletal disorders in the elderly is still scarce. Future healthcare research focusing on this age group should be a priority in developing countries since their public healthcare systems are not yet fully prepared to accommodate the needs of an aging population.

## **Background**

Population ageing is a worldwide phenomenon caused by the reductions in adult mortality and fertility. The latter is the primary cause of population ageing given that sustained fertility reductions lead to an increase in the proportion of older age groups [1]. This demographic change was first observed in developed countries in the nineteenth century and more recently has been transforming the societies of developing and low-income countries. In Brazil, fertility rates dropped by 60% between 1970 and 2000 [2]. The latest population census conducted in 2010 identified over 20.5 million elderly Brazilians [3]. By 2050, it is expected that this number reaches 64 million, placing Brazil as the fifth nation with the greatest number of older people [1].

With population ageing, the most prevalent types of diseases shift from acute infectious to chronic non-communicable diseases, such as chronic musculoskeletal conditions. In the most recent Brazilian National Household Survey, around 80% of Brazilians aged 60 years or more reported having at least one chronic non-communicable disease, with chronic musculoskeletal disorders being the most prevalent group of diseases (including spine problems, osteoarthritis and rheumatoid arthritis) [4].

Pain is the primary complaint of individuals with chronic musculoskeletal disorders [5], and it is particularly important in the elderly because of its impact on quality of life, independence and social participation. The economic burden of musculoskeletal pain is enormous, being only lower to that caused by cardiovascular disease [6]. Most individuals will present with “nonspecific pain”, a condition in which it is not possible to identify a single specific cause for the pain, even when pain is restricted to one location (e.g. lower back) [7]. This situation is challenging to healthcare practitioners since it requires a more complex approach to diagnosis and treatment. Conversely, a specific diagnosis is possible for some chronic musculoskeletal disorders, such as osteoarthritis. However, regardless of whether a specific diagnosis is possible, the presence of co-morbidities in the elderly makes pain management even more challenging.

The high prevalence of chronic musculoskeletal disorders along with population ageing is worrisome. This is particularly problematic in developing and low-income countries, where the society and the public healthcare system are not yet fully prepared to fulfill the needs required by this recent scenario. Knowing the problem in details is essential for the development of appropriate health policies that incorporate a strategic plan for the promotion of health and prevention of disabilities in the elderly population. This systematic review investigated the prevalence of chronic musculoskeletal disorders in elderly Brazilians.

## **Methods**

### **Search strategy**

The following electronic databases were searched from inception to January 2012: MEDLINE, LILACS, SCIELO, Brazilian Digital Library of Theses and Dissertations (BDTD) and CAPES/MEC Theses Database. The search terms and combinations used for MEDLINE were ((musculoskeletal diseases OR rheumatic diseases OR rheumatology OR arthritis OR osteoarthritis) AND prevalence AND (elder* OR aging OR aged OR geriatric*) AND (cross-sectional OR survey) AND Brazil). The search strategies for the other databases are available upon request. We also hand searched reference lists of relevant reviews and primary studies. Our searches did not have any language restrictions.

### **Study selection**

Study selection was performed by two independent review authors, and a third review author was consulted to solve disagreements. Eligibility was first assessed through the screening of titles and abstracts, and the full text of all potentially eligible papers was retrieved to confirm eligibility.

Cross-sectional studies reporting on the prevalence of chronic musculoskeletal disorders in elderly Brazilians were eligible for inclusion. Among the numerous diagnoses considered within the group of chronic musculoskeletal disorders, it was decided *a priori* that all were to be included except for temporomandibular joint (TMJ) disorders, rheumatoid arthritis, systemic lupus erythematosus and osteoporosis. These diagnoses were excluded because of the particularities of their underlying mechanisms and clinical presentations.

To determine whether the study reported on chronic musculoskeletal disorders, we relied upon information presented in the manuscript title, text or tables. For the definition of elderly, we followed the recommendation of the Department of Economic and Social Affairs of the United Nations (UN), which considers as elders those individuals with 60 years of age or older [1]. Studies reporting on the prevalence of musculoskeletal disorders in various age groups were considered for inclusion only when it was possible to extract prevalence data in the elderly population.

### **Data extraction and quality assessment**

Two review authors independently extracted data on study characteristics (design, location, measure of prevalence), participants’ characteristics and rates of chronic musculoskeletal disorders. One review author extracted data to evaluate the methodological quality of studies. Internal validity was assessed according to the following criteria [8]: (1) adequacy of sampling (random sample); (2) sample size calculation; (3) sufficient response rate (> 80%); (4) low potential for recall bias (assessment of present chronic pain instead of past chronic pain); (5) use of a validated measurement tool or physical examination by a doctor/physiotherapist to ascertain chronic musculoskeletal disorders. Study quality was considered low if at least 3/5 quality criteria were not met.

### **Data synthesis**

Results of the included studies were grouped into the diagnoses “chronic musculoskeletal pain” or “specific musculoskeletal diagnoses”, and a critical synthesis of the results was performed.

## **Results**

**Figure 1:**
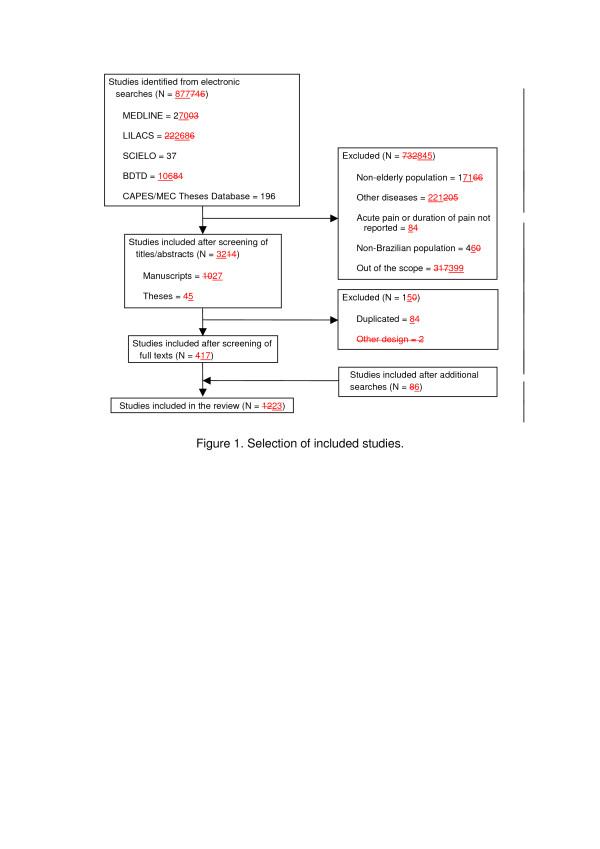
**Selection of included studies.** * Three studies were reported in one publication.

The electronic search retrieved 877 potentially eligible studies: 270 in MEDLINE, 268 in LILACS, 37 in SCIELO, 106 in BDTD and 196 in CAPES/MEC Theses Database. After screening of full texts, a total of 17 studies fulfilled the inclusion criteria and were included [5,9-24]. Six additional studies found after screening of reference lists were included [25-30]. Figure 1 describes the selection of the 23 included studies.

The full text of two studies [26,30] could not be retrieved and relevant data were extracted from their abstracts. One of the included studies reported on the results of the Brazilian National Household Surveys conducted in 1998 [16]a], 2003 [16]b] and 2008 [16]c]. Results from each of these surveys were considered separately in this review, totalizing 25 included studies. The included studies reported on a total of 116,091 elderly Brazilians, with individual samples varying from 25 [25] to 41,269 [16]c] participants.

Brazil is a Federative Republic made up of 26 Federation Units called States and the Federal District (Brasilia). Except for the Brazilian National Household Surveys, which recruited participants from all Federation Units [16]a, [16]b, [16]c], the included studies were conducted in seven different States: Bahia [12,13,21,25], Goiás [28], Minas Gerais [14,18,23], Paraná [5,11,20,29,30], Rio Grande do Sul [24,26], Santa Catarina [17,27] and São Paulo [9,10,15,19,22]. Eight studies were conducted in capital cities with population ranging from 421,000 (Florianópolis [27]) to 11.3 million inhabitants (São Paulo [19,22]) [31].

Most study samples included community-dwelling elders, predominantly women in the age group of 60 to 69 years old. Three studies [10,12,27] recruited elders seeking care from a healthcare practitioner, and one [13] recruited institutionalized elders. One study recruited both institutionalized elders and elders seeking care in an Emergency Department [20].

The procedure used for data collection and the measure of prevalence of the included studies are listed in Table 1. One study [12] collected prevalence data from medical records. Two studies [13,26] used validated questionnaires to evaluate the presence and quality of chronic pain. In six studies the musculoskeletal diagnosis was given or confirmed by a healthcare practitioner after clinical or radiological examination [9,10,20,22,23,27]. In all other studies pain was evaluated by single questions about its presence, location and intensity [5,11,14-19,21,24,25,28-30].

**Table 1:** Measures of prevalence of chronic musculoskeletal disorders

**Study**	**Measure of prevalence**
**Alves**[26]	In a face-to-face interview, participants filled in the Portuguese-Brazil version of the Nordic Musculoskeletal Questionnaire [32], which assessed the presence of pain or discomfort in a specific anatomical area in the past 12 months and in the past week (including present pain), and if the pain interfered with activities of daily living.
**Backer**[27]	Health care practitioner followed the ACR criteria to diagnose knee OA by clinical examination.
**Cassettari**[9]	In a face-to-face interview, participants (or caregiver) were asked if they had knee pain. Participants reporting knee pain were examined by a rheumatologist to confirm the clinical diagnosis of OA.
**Coimbra**[10]	Medical diagnosis of OA by clinical examination. Details of the examination were not described. Diagnosis of hand OA was confirmed by x-ray.
**Dellaroza**[5]	In a face-to-face interview, participants were asked about the presence of musculoskeletal pain lasting for 6 months or more (continuous or recurrent pain) in the past 12 months, its location, frequency and intensity.
**Dellaroza**[11]	In a face-to-face interview, participants were asked about the presence of musculoskeletal pain lasting for 6 months or more (continuous or recurrent pain), its location, frequency and intensity.
**dosReis**[12]	Medical records were screened and data were extracted regarding musculoskeletal diagnoses and main complaints.
**dosReis**[13]	In a face-to-face interview, participants filled in the McGill Pain Questionnaire [33], which assessed pain location, pain descriptors and intensity.
**Giacomin**[14]	In a face-to-face interview, participants (or another household member or caregiver) were asked if they had ever received the diagnosis of arthritis by a doctor or other healthcare professional.
**Lacerda**[28]	In a face-to-face interview, participants were asked about the presence of chronic pain, defined as "an unpleasant sensorial or emotional experience, constant or recurrent, which end cannot be foreseen, lasting for more than 6 months", its location and intensity.
**Lima**[15]	In a face-to-face interview, participants were asked if they had one of the following musculoskeletal problems: arthritis, rheumatism and back pain.
**Lima-Costa**[16]a]	In a face-to-face interview, participants (or another household member or caregiver) were asked if they had arthritis or rheumatism.
**Lima-Costa**[16]b]	In a face-to-face interview, participants (or another household member or caregiver) were asked if they had ever received the diagnosis of arthritis or rheumatism by a doctor or other healthcare professional.
**Lima-Costa**[16]c]	Same as above.
**Liposcki**[17]	In a face-to-face interview, participants were asked if they had OA.
**Machado**[18]	In a face-to-face interview, participants (or another household member or caregiver) were asked if they had ever been diagnosed with rheumatism by a doctor and the level of associated disability, and if they had ever presented with pain (including aching and stiffness) in the hands or knees on most days for at least 6 weeks. These questions were copied from the Third National Health and Nutrition Examination Survey [34] and translated by the authors to Portuguese-Brazil.
**Menéndez**[19]	In a face-to-face interview, participants were asked if they had arthritis.
**Panazzolo**[29]	In a face-to-face interview, participants were asked about the presence of musculoskeletal pain lasting for 6 months or more (continuous or recurrent pain) and its location.
**Panazzolo**[30]	In a face-to-face interview, participants were asked about the presence of musculoskeletal pain lasting for 6 months or more (continuous or recurrent pain), its location, frequency and intensity, and if pain interfered with function, sleep or mood.
**Rey**[20]	Assessor followed the ACR criteria to diagnose hand OA by clinical examination.
**Sá**[21]	In a face-to-face interview, participants were asked about the presence of chronic pain, defined as pain felt more than one time at the same body region for over 6 months, and its location.
**Sampaio**[25]	In a face-to-face interview, participants were asked about the presence of chronic pain and pre-existing musculoskeletal pathologies.
**Santos**[22]	Chronic widespread pain was diagnosed by two trained researchers if the participant presented with diffuse pain in the axial skeleton on both sides of the body, above and beyond the hip, for more than 3 months with less than 11 positive tender points (assessed using a Fischer dolorimeter). Fibromyalgia was diagnosed by a rheumatologist according to the ACR criteria.
**Senna**[23]	In a face-to-face interview, participants were asked about the presence of pain or tenderness in bones, joints, or muscles in the last 7 days that was not related to trauma. Those answering “yes” were examined by a rheumatologist to confirm the diagnosis of OA or fibromyalgia.
**Silval**[24]	In a face-to-face interview, participants were asked about the presence of low back pain for at least 7 weeks.

Legend: *OA* osteoarthritis; *ACR* American College of Rheumatology.

According to the study reports, chronic pain was considered pain that persisted for six weeks or more [18], seven weeks or more [24], three months or more [22], or six months or more [5,11,21,28-30]. In 12 of the included studies, authors did not make clear what was their definition for chronic symptoms [12-17,19,23,25,26].

Most studies (72.0%) investigated the report of present musculoskeletal disorders [9-13,15,16]a,[17,19-22,24,25,27-30], whereas other studies provided prevalence data based on recall periods that ranged from one week [23,26] to lifetime [14,16]b, [16]c, [18]. Six studies [9,14,16]a, [16]b, [16]c, [18] allowed the response to be reported by proxy (either another household member or caregiver).

Eight studies (32.0%) were of high methodological quality [9,10,15,16]a,[21-24]. Only one study fulfilled all the quality criteria [23]. Quality ratings for each study are described in Table 2.

**Table 2:** Methodological quality of included studies

**Study**	**Quality criteria**				
	**1**	**2**	**3**	**4**	**5**
**Alves**[26]	-	-	?	+	+
**Backer**[27]	-	-	?	+	+
**Cassettari**[9]	+	-	?	+	+
**Coimbra**[10]	-	+	?	+	+
**Dellaroza**[5]	-	-	+	-	-
**Dellaroza**[11]	-	-	-	+	-
**dosReis**[12]	-	-	?	+	+
**dosReis**[13]	-	-	?	+	+
**Giacomin**[14]	+	+	?	**-**	**-**
**Lacerda**[28]	-	-	-	+	-
**Lima**[15]	+	+	?	+	-
**Lima-Costa**[16]a]	+	+	?	+	-
**Lima-Costa**[16]b]	+	+	?	-	-
**Lima-Costa**[16]c]	+	+	?	-	-
**Liposcki**[17]	-	-	?	+	-
**Machado**[18]	-	-	+	-	-
**Menéndez**[19]	+	-	?	+	-
**Panazzolo**[29]	-	-	?	+	-
**Panazzolo**[30]	-	-	?	+	-
**Rey**[20]	**-**	**-**	?	+	+
**Sá**[21]	+	+	+	**-**	**-**
**Sampaio**[25]	-	-	?	+	-
**Santos**[22]	+	+	?	+	+
**Senna**[23]	+	+	+	+	+
**Silval**[24]	+	+	+	+	-

Legend: + = criterion was met; - = criterion was not met; ? = uncertain. (1) adequacy of sampling; (2) sample size calculation; (3) sufficient response rate; (4) low potential for recall bias; (5) use of a validated measurement to ascertain chronic musculoskeletal disorders.

### **Prevalence of chronic musculoskeletal disorders**

The main results on the prevalence of chronic musculoskeletal disorders are presented in Table 3.

**Table 3:** Characteristics of included studies and results

**Study**	**City (State)**	**Participants**	**Prevalence of chronic musculoskeletal disorders**
**Alves**[26]	Porto Alegre (Rio Grande do Sul)	57 community-dwelling elders who performed routine physical activity: mean age 68.5 ± 5.7; 71.9% F.	CMP in the past 12 months: low back pain (40.4%); neck and shoulder pain (35.1%); knee pain (33.3%).
			CMP in the past 12 months that interfered with ADLs: elbow pain (33.3%); low back pain (30.4%); shoulder pain (25.0%); wrist/hand and thoracic pain (20.0%).
			CMP in the past week: thoracic pain (80.0%); elbow pain (66.7%); low back pain (65.2%); hip/thigh and neck pain (50.0%).
**Backer**[27]	Florianópolis (Santa Catarina)	62 elders 60 years old or more*, who were seeking care from a local healthcare unit.	Specific diagnoses: knee OA (37.5%). Prevalence of knee OA was positively associated with age (p < 0.01) and BMI (p < 0.05).
**Cassetari**[9]	Botucatu (São Paulo)	355 community-dwelling elders 60 years old or more: 35.3% F.	CMP: knee pain (64.0%). Specific diagnosis: knee OA (20.6%).
**Coimbra**[10]	Campinas (São Paulo)	106 elders 60 years old or more*, referred to a rheumatology triage center.	Specific diagnoses: hand OA (28.3%); OA in other location (31.1%). Prevalence of hand OA was positively associated with BMI (OR 1.05; 95% CI 1.00 - 1.11).
**Dellaroza**[5]	Londrina (Paraná)	451 elderly municipal employees 60 years old or more: 35.3% F.	CMP: pain in any location (51.4%); spinal pain (21.7%); lower limb pain (21.5%); headache (7.1%); upper limb pain (4.4%); neck pain (1.3%); pelvic pain (0.7%); other (8.4%).
			Most participants reported daily intermittent CMP of low intensity that was not triggered during a specific time of the day. For those with multiple pain sites, lower limb pain was the most bothering, followed by spinal pain.
**Dellaroza**[11]	Londrina (Paraná)	172 community-dwelling elders 60 years old or more who had frequent pain complaints: 58.7% F.	CMP: pain in any location (62.2%); lower limb pain (31.4%); spinal pain (30.2%); shoulder and upper limb pain (11.1%); headache (7.6%); generalized pain (4.7%); neck pain (3.4%).
			Most participants reported daily intermittent CMP of low intensity that was not triggered during a specific time of the day. Prevalence of CMP was positively associated with age (p = 0.02), female gender (p < 0.01) and depression (p < 0.01).
**dosReis**[12]	Jequié (Bahia)	131 elders 60 years old or more who were under physiotherapy treatment in a university clinic: 65.6% F.	CMP: low back pain (15.3%); neck pain (6.9%); joint pain (6.1%). Specific diagnoses: OA (33.6%); fracture (9.2%); tendinitis (9.2%); herniated disc (6.1%); bursitis (3.8%); other (10.7%).
			Main complaint: pain (85.5%); reduction in mobility (9.9%); paresthesia (8.4%); weakness (6.1%); gait disturbance (6.1%); stiffness (1.5%).
**dosReis**[13]	Jequié (Bahia)	60 institutionalized elders 60 years old or more who did not present cognitive deficit: mean age 77.6 ± 11.6; 50.0% F.	CMP: pain in any location (73.3%); spinal pain (31.0%); lower limb pain (28.2%); upper limb pain (14.1%); location not mentioned (2.8%).
			Pain intensity: light (52.3%); moderate (34.1%); intense (13.6%). Most common pain descriptors: shooting and stabbing (sensory pain group); annoying and unbearable (affective pain group); miserable and nauseating (evaluative pain group); tiresome and tightening (miscellaneous pain group).
			Prevalence of CMP was higher among elders with cognitive deficit (p < 0.01).
**Giacomin**[14]	Belo Horizonte (Minas Gerais)	1,786 community-dwelling elders 60 years old or more, who participated in a health survey conducted in 2003: mean age 69.7 ± 9.1; 58.9% F.	Specific diagnoses: OA (16.6%). The prevalence of OA was associated with moderate difficulty in performing ADLs (OR 2.01; 95% CI 1.24 - 3.25).
**Lacerda**[28]	Goiânia (Goiás)	40 elders 60 years old or more covered by a team of the Family Health Program: 60–70 years old (57.5%), 71–80 years old (30.0%), 81–86 years old (12.5%); 67.5% F.	CMP: pain in any location (62.5%); spinal pain (48.0%); lower limb pain (24.0%); headache (8.0%); pain on the right side of the body (8.0%); pain on the anterior thorax (8.0%); upper limb pain (4.0%).
			Pain intensity: light (28.0%); moderate (20.0%); intense (16.0%); unbearable (36.0%).
			A large proportion of participants with CMP reported functional disability (68.0%), fatigue (52.0%), sleeping disturbance (48.0%), irritability (44.0%), fear of a new lesion (32.0%), depression (28.0%) and agitation (28.0%).
**Lima**[15]	Botucatu, Campinas, Itapecerica da Serra, Embu, Taboão da Serra, São Paulo (São Paulo)	1,958 community-dwelling elders 60 years old or more, who participated in the 2001–2002 ISA-SP Study: mean age 69.9 ± 0.4; 57.2% F.	CMP: back pain (30.1%). Specific diagnoses: arthritis or rheumatism (27.2%).
**Lima-Costa**[16]a]	All Federation Units and Federal District	28,943 community-dwelling elders 60 years old or more, who participated in the 1998 PNAD study: mean age 69.5 (95% IC 69.4 - 69.6); 55.5% F.	Specific diagnoses: arthritis or rheumatism (37.5%; 95% CI 35.4% - 40.0%)^†^.
**Lima-Costa**[16]b]	Same as above	35,042 community-dwelling elders 60 years old or more, who participated in the 2003 PNAD study: mean age 69.8 (95% IC 69.5 - 69.9); 55.9% F.	Specific diagnoses: arthritis or rheumatism (27.3%; 95% CI 25.4% - 29.2%)^†^.
			Prevalence decreased from 1998 to 2003 (PR 0.72; 95% CI 0.70 - 0.75).
**Lima-Costa**[16]c]	Same as above	41,269 community-dwelling elders 60 years old or more, who participated in the 2008 PNAD study: mean age 69.9 (95% IC 69.8 - 70.0); 56.2% F.	Specific diagnoses: arthritis or rheumatism (24.2%).
			Prevalence decreased from 1998 to 2008 (PR 0.64; 95% CI 0.62 - 0.66).
**Liposcki**[17]	Lages (Santa Catarina)	101 elders 60 years old or more: mean age 77.1 (range 60–106); 62.4% F.	Specific diagnoses: OA (39.6%). Self-report of OA was associated with the report of falls in the previous 6 months (p = 0.02).
**Machado**[18]	Bambuí (Minas Gerais)	1,606 community-dwelling elders 60 years old or more: 60.1% F.	CMP: hand and knee pain (44.2%). Specific diagnoses: arthritis or rheumatism (25.3%).
			Prevalence of CMP was lower among men (OR 0.56; 95% CI 0.46 - 0.69), elders with 8 or more years of study** (OR 0.50; 95% CI 0.33 - 0.75), with income of at least 10 times the minimum wage^††^ (OR 0.60; 95% CI 0.40 - 0.90) and current smokers^‡‡^ (OR 0.66; 95% CI 0.50 - 0.87). Prevalence of CMP was higher among elders with BMI from 30 to 34 Kg/m^2^ (OR 3.07; 95% CI 1.97 - 4.80)*** and those reporting previous myocardial infarct (OR 2.26; 95% CI 1.39 - 3.67), cerebrovascular accident (OR 4.32; 95% CI 2.35 - 7.93), Chagas disease (OR 1.79; 95% CI 1.42 - 2.27) and diabetes (OR 1.43; 95% CI 1.07 - 1.90).
			Prevalence of specific diagnoses was lower among men (OR 0.38; 95% CI 0.30 - 0.50) and previous smokers^‡‡^ (OR 0.67; 95% CI 0.50 - 0.90). Prevalence of specific diagnoses was higher among elders with BMI from 30 to 34 Kg/m^2^ (OR 2.39; 95% CI 1.47 - 3.88)***, with cholesterol levels from 200 to 239 m% (OR 1.45; 95% CI 1.06 - 1.98)^†††^ and those reporting previous myocardial infarct (OR 1.74; 95% CI 1.07 - 2.84), cerebrovascular accident (OR1.75; 95% CI 1.02 - 3.00) and Chagas disease (OR 1.33; 95% CI 1.03 - 1.73).
**Menéndez**[19]	São Paulo (São Paulo) and 6 other cities in Latin America and the Caribbean	2,143 community-dwelling elders 60 years old or more^‡^: mean age 73.3; 58.9% F.	Specific diagnoses: OA (32.8%). Prevalence of OA was associated with the difficulty in performing ADLs and IADLs (p < 0.01).
**Panazzolo**[29]	Londrina (Paraná)	245 community-dwelling elders 60 years old or more: mean age 68.8 ± 6.9; 57.6% F.	CMP: pain in any location (67.7%); lower limb pain (42.0%); low back pain (27.8%).
**Panazzolo**[30]	Londrina (Paraná)	111 community-dwelling elders 60 years old or more: mean age 70.1 ± 7.5; 65.8% F.	CMP: lower limb pain (52.3%); spinal pain (48.6%).
			Most participants with lower limb pain reported daily intermittent episodes. Most spinal pain was of high intensity (75.9%). CMP was associated with difficulties in performing the following functional tasks: walk near home (p < 0.01); get in and out of bed (p < 0.05); travel (p < 0.01); shop (p < 0.01); cook own meal (p < 0.01); domestic chores (p < 0.01); take care of own money (p = 0.05).
			Pain interfered with sleep and mood in 61.3% and 55.0% of participants, respectively.
**Rey**[20]	Curitiba (Paraná)	239 elders 60 years old or more*, who lived in long-term care institutions or who sought care at an Emergency Department of a University Hospital or at a Basic Healthcare Unit.	Specific diagnoses: hand OA (14.2%). Prevalence of OA was associated with female gender (p < 0.01).
**Sá**[21]	Salvador (Bahia)	197 community-dwelling elders 65 years old or more*, who participated in the MONIT Study: 62.4% F.	CMP: pain in any location (56.3%).
			CMP was associated with female gender (p < 0.05). Prevalence of CMP was lower among female elders reporting moderate alcohol consumption^‡‡‡^ (adjusted OR 0.74; 95% CI 0.57 - 0.97) and single male elders**** (adjusted OR 0.68; 95% CI 0.47 - 0.98). Prevalence of CMP was higher among female elders reporting excessive alcohol consumption^‡‡‡^ (adjusted OR 7.11; 95% CI 1.59 - 31.82), previous female and male smokers^‡‡^ (adjusted OR 1.41; 95% CI 1.02 - 1.96 and 1.78; 95% CI 1.22 - 2.59, respectively) and current male smokers^‡‡^ (adjusted OR 1.45; 95% CI 1.04 - 2.02).
**Sampaio**[25]	Jequié (Bahia)	25 community-dwelling elders 60 years old or more who took part in a third age relationship group: 60.0% F.	CMP: pain in any location (77.7%); lower limb pain (21.0%); low back pain (20.0%); shoulder pain (20.0%); upper limb pain (11.0%); neck pain (7.0%); hip pain (1.0%).
			Specific diagnoses: herniated disc (26.9%); fracture (18.1%); bursitis (16.9%); tendinitis (14.4%); OA (9.4%); chondromalacia (9.4%).
**Santos**[22]	São Paulo (São Paulo)	361 community-dwelling elders 65 years old or more: mean age 73.3 ± 5.7; 64.0% F.	CMP: chronic widespread pain (14.1%; 95% CI 10.5% - 17.7%).
			Specific diagnoses: fibromyalgia (5.5%; 95% CI 5.4% - 5.7%).
			CMP was associated with female gender (p < 0.01) and fewer years of education (p < 0.05).
			Participants with fibromyalgia had higher BMI than those without pain (p < 0.05) and lower pain threshold (p < 0.001), higher fatigue, tiredness, stiffness and impact on work than those with chronic widespread pain (p < 0.05). Participants with fibromyalgia and chronic widespread pain had higher anxiety scores than those without pain (p < 0.05).
**Senna**[23]	Montes Claros (Minas Gerais)	48 community-dwelling elders 75 years old or more.	Specific diagnoses: OA (22.9%; 95% CI 11.1% - 34.9%); fibromyalgia (0%).
**Silva**[24]	Pelotas (Rio Grande do Sul)	583 community-dwelling elders 60 years old or more.	CMP: low back pain (5.1%).

Legend: *F* Female; *CMP* chronic musculoskeletal pain; *OA* osteoarthritis; *BMI* body mass index; *ADL* activities of daily living; *IADLs* instrumental activities of daily living; *ISA-SP* São Paulo State Health Survey; *PNAD* National Household Survey; *MONIT* Project Monitoring Cardiovascular Diseases and Diabetes; *CI* confidence interval; *OR* odds ratio *PR* prevalence ratio. Mean age ± standard deviation were presented when available in the study report. *Data on elders were extracted from graph or table provided by authors. ^†^CI retrieved from a previous publication. ^‡^Data on Brazilian participants extracted from table provided by authors. **Compared with illiterate elders. ^††^Compared with elders with income < 2 times the minimum wage. ^‡‡^Compared with non-smokers. ***Compared with elders with BMI < 20 Kg/m^2^. ^†††^Compared with elders with cholesterol < 200 m%. ^‡‡‡^Compared with non-drinkers. ****Compared with married male elders.

### **Musculoskeletal pain**

Fifteen studies estimated the prevalence of chronic musculoskeletal pain [5,9,11-13,15,18,21,22,24-26,28-30]. Studies reporting on chronic musculoskeletal pain in any location found estimates ranging from 14.1% [22] to 85.5% [12]. Among the studies evaluating multiple pain sites, the spine (50.0%) [5,13,26,28] and the lower limb (50.0%) [11,25,29,30] were the most prevalent pain locations. Dellaroza et al. [5] found that when the spine is the most prevalent pain site, elders consider the concomitant lower limb pain as the most bothering pain. Prevalence estimates for lower limb pain and spinal pain ranged from 21.0% [25] to 64.0% [9], and from 5.1% [24] to 65.2% [26], respectively.

Five studies investigated pain intensity [5,11,13,28,30] and in most of them elders reported having low levels of chronic musculoskeletal pain [5,11,13]. In the studies conducted by Lacerda et al. [28] and Panazzolo et al. [30], most participants reported high to unbearable pain. Both studies also found pain interference with functional tasks, sleep and mood in a large proportion of elders. Nearly one third of elders from the study of Lacerda et al. [28] feared a new lesion (Table 3).

### **Specific musculoskeletal diagnoses**

Sixteen studies (64.0%) estimated the prevalence of specific musculoskeletal diagnoses [9,10,12,14-20,22,23,25,27]. In seven studies the specific diagnosis was given by a healthcare professional [9,10,12,20,22,23,27], whereas other studies used participants’ self-report on whether they had the disease or had been given a diagnosis by a doctor or other healthcare professional.

The studies generally reported prevalence estimates for arthritis and rheumatism in general (including osteoarthritis), with prevalence estimates ranging from 9.4% [25] to 39.6% [17]. Four studies investigated the presence of osteoarthritis in specific body sites (hand [10,20] and knee [9,27]). Prevalence estimates ranged from 14.2% to 28.3% for hand osteoarthritis and from 20.6% to 37.5% for knee osteoarthritis.

Four studies [12,22,23,25] investigated the prevalence of other musculoskeletal diagnoses and found the following prevalence estimates: 3.8% to 16.9% for bursitis, 6.1% to 26.9% for herniated disc, 9.2% to 18.1% for fracture, 9.2% to 14.4% for tendinitis, 9.4% for chondromalacia and 0.0% to 5.5% for fibromyalgia.

### **Chronic musculoskeletal disorders in different patient groups**

Twelve studies [10,11,13,14,17-22,27,30] investigated the association of the presence of chronic musculoskeletal disorders with a wide range of factors. Studies reported statistically significant associations between chronic musculoskeletal disorders and older age [11,27], female gender [11,18,20-22], married status [21], cognitive deficit [13], current or previous smoking [18,21], report of falls [17] and co-morbidities [18]; lower education [18,22], lower income [18], lower functional capacity [14,19,30], lower pain threshold [22]; higher BMI [10,18,22,27], excessive alcohol consumption [21], work impact [22], fatigue [22], tiredness [22], stiffness [22], depression [11] and anxiety [22]. These associations are described in details in Table 3.

## **Discussion**

To our knowledge this was the first systematic review to synthesize the results of studies investigating the prevalence of chronic musculoskeletal disorders in elderly Brazilians. Evidence from the 23 reports (25 included studies) indicated that these disorders affect an important part of the elderly population, with estimates reaching 85.5% for chronic musculoskeletal pain in any location.

The most representative prevalence estimates come from the Brazilian National Household Surveys conducted in 1998, 2003 and 2008, which present data from over 105,200 community-dwelling elders living in every Brazilian State and the Federal District. The use of such broad samples are important in epidemiological research of continental-sized countries like Brazil, where the socio-demographic characteristics of the population are largely variable across the national territory; for example, the Human Development Index (HDI) of Bahia is 0.59, whereas the HDI of Rio Grande do Sul is 0.75 [35].

Interestingly, the prevalence of chronic musculoskeletal disorders has decreased between the Brazilian National Household Surveys conducted in 1998 and 2003 and between the surveys conducted in 1998 and 2008, but not between the latest two occasions of the national survey. This has been attributed to changes in how prevalence was measured in these surveys [16].

The definitions of chronic musculoskeletal pain were largely variable among the included studies. According to the International Association for the Study of Pain (IASP), chronic pain is defined as an episode of pain of at least six months [6]. Specifically for chronic low back pain, the most recent clinical practice guidelines define as chronic an episode of low back pain of at least 12 weeks [36]. In most studies included in this review, the definitions of chronic pain did not reflect these recommendations or the authors did not offer sufficient information to judge. The inconsistency among definitions is a problem given that it limits the interpretation and comparison among study results on this topic.

Two previous systematic reviews investigated the prevalence of low back pain in the elderly [37,38], but none of them included the studies conducted in Brazil. Bressler et al. [37] found prevalence estimates for low back pain ranging from 12.8% to 49.0% among community-dwelling elders. According to our results, the prevalence of low back pain in elderly Brazilians ranged from 5.1% to 65.2%. This large variation in prevalence estimates may be due to a number of factors, which include different definitions of chronic symptoms, recall bias and proxy reporting.

The review of Dionne et al. [38] reported the presence of a linear relationship between severe low back pain and age, but not between benign low back pain and age. The positive association between chronic musculoskeletal disorders and age was also reported in two studies included in the present review [11,27]. In the study of Dellaroza et al. [11], this association was present among elders reporting low levels of pain.

Low back pain is currently listed as the most prevalent musculoskeletal disorder among adults in the world [39]. Nevertheless, our results indicate that the prevalence of low back pain is similar to the prevalence of lower limb pain in elderly Brazilians. This finding is comparable to that of Urwin et al. [40], who investigated the prevalence of musculoskeletal disorders in 5,000 individuals from Manchester (UK). The authors found that low back pain was the most prevalent musculoskeletal disorder in individuals younger than 65 years old, whereas knee pain was the most prevalent condition in those aged 65 years or more, with a peak in women aged 75 years and older [40].

It is possible that the differences in self-reported prevalence estimates among the various age groups would reflect extrinsic factors not related to an actual dissimilar distribution of chronic musculoskeletal pain. One factor would be related to the disability (or its perception by the individual) associated with chronic pain. If this is the case, low back pain may be the most prevalent musculoskeletal disorder among elders, but lower limb pain is more frequently reported by them given its greater impact on function, including gait impairments and increased risk of falls. It is also possible that elders consider low back pain as a trivial and less important condition because they may have experienced many low back pain episodes throughout the lifespan that did not lead to any serious consequences to their health.

The presence of co-morbidities, which are common in elders, can also affect their perception of pain. Dellaroza et al. [11] found a significant increase in the report of chronic musculoskeletal pain among depressive elders. A number of recently published studies provide evidence to support the relationship between chronic musculoskeletal pain and depression [41-43]. Interestingly, our findings reflect the importance not only of co-morbidities that directly affect the perception of pain (i.e. depression, anxiety, cognitive deficit), but also co-morbidities that may impact the perception or the report of pain in elders by indirect mechanisms. Some co-morbidities found to be associated with chronic musculoskeletal pain in this review include cardiovascular diseases and diabetes (Table 3).

The diagnosis of osteoarthritis is strongly associated with ageing, irrespective of the location (small joints or large weight bearing joints) or gender [44]. Among the specific musculoskeletal diagnoses investigated by the studies included in this review, the broad group of arthritis and rheumatism (including osteoarthritis) was the most prevalent, followed by the diagnosis of herniated disc. In 2004, the World Health Organization (WHO) estimated that over 150 million individuals had osteoarthritis in the world and that this condition was the fifth and ninth cause of years lost due to disability (YLD) in low/middle-income and high-income countries, respectively [45]. Moreover, recent data indicate a staggering 30% increase in the prevalence of this condition in one decade [46]. This large increase is due to a combination of factors, which include ageing of the population, rising prevalence of risk factors (i.e. obesity), and the increased use of imaging [46,47].

We found higher self-reported prevalence rates of chronic musculoskeletal disorders among elderly women. This result is in line with the literature and may be related to fact that women are better at perceiving their physical signs and symptoms than men, and to the knowledge acquired from their role as the family caregiver [48]. Additionally, women may have a higher risk of developing musculoskeletal problems due to anatomo-functional particularities such as shorter height, lower muscle mass and bone mineral density, increased joint laxity and lower degree of adaptation to physical effort [24,49].

The number of epidemiological studies investigating the prevalence of chronic musculoskeletal disorders among elders is still limited, particularly in developing and low-income countries. This reflects the socioeconomic demand for research focusing on the working population. Along with the limited number of studies targeting the elderly population, the low quality of the existing studies makes the interpretation of the evidence still more difficult; for example, only one third of the studies included in this review were of high quality.

With the growth of the elderly population in the developing world, future high-quality research focusing on this age group is mandatory in order to clarify the health needs of this population and to plan necessary changes in the public healthcare system.

## **Conclusions**

Brazil will soon be the fifth nation with the greatest number of older people in the world. However, high-quality epidemiological research on chronic musculoskeletal disorders in elderly Brazilians is still limited. The results of this review indicate that chronic musculoskeletal disorders are highly prevalent among elderly Brazilians and should therefore be considered in future public healthcare policies targeting this age group.

## **Competing interests**

The authors declare that they have no competing interests.

## **Authors’ contributions**

VM, LM and JD participated in the conception and design of the study. VM and VdC performed the literature search and selection of studies. VM, VdC and LM extracted relevant data. VM, LM and JD participated in the analysis and interpretation of data and in the preparation and revision of this manuscript. All authors read and approved the final manuscript.

## Pre-publication history

The pre-publication history for this paper can be accessed here:

http://www.biomedcentral.com/1471-2474/13/82/prepub
